# Offering patients a choice for colorectal cancer screening: a quality improvement pilot study in a quality circle of primary care physicians

**DOI:** 10.1136/bmjoq-2019-000670

**Published:** 2019-10-03

**Authors:** Yonas Martin, Leo Alexander Braun, Marc-Andrea Janggen, Kali Tal, Nikola Biller-Andorno, Cyril Ducros, Kevin Selby, Reto Auer, Adrian Rohrbasser

**Affiliations:** 1 Institute of Primary Health Care (BIHAM), University of Bern, Bern, Switzerland; 2 Department of General Internal Medicine, Inselspital, Bern University Hospital, University of Bern, Bern, Switzerland; 3 Institute for Biomedical Ethics and History of Medicine (IBME), University of Zurich, Zurich, Switzerland; 4 Foundation for Cancer Screening of the Canton of Vaud (FVDC), Lausanne, Switzerland; 5 Center for Primary Care and Public Health (Unisanté), University of Lausanne, Lausanne, Switzerland; 6 Medbase, Wil, Switzerland

**Keywords:** primary care, shared decision making, quality improvement, patient-centred care, PDSA

## Abstract

**Background:**

Guidelines recommend primary care physicians (PCPs) offer patients a choice between colonoscopy and faecal immunochemical test (FIT) for colorectal cancer (CRC) screening. Patients choose almost evenly between both tests but in Switzerland, most are tested with colonoscopy while screening rates are low. A quality circle (QC) of PCPs is an ideal site to train physicians in shared decision-making (SDM) that will help more patients decide if they want to be tested and choose the test they prefer.

**Objective:**

Systematically assess CRC screening status of eligible 50–75 y.o. patients and through SDM increase the proportion of patients who have the opportunity to choose CRC screening and the test (FIT or colonoscopy).

**Methods:**

Working through four Plan-Do-Study-Act (PDSA) cycles in their QC, PCPs adapted tools for SDM and surmounted organisational barriers by involving practice assistants. Each PCP included 20, then 40 consecutive 50–75 y.o. patients, repeatedly reported CRC status as well as the proportion of eligible patients with whom CRC screening could be discussed and patients’ decisions.

**Results:**

9 PCPs initially included 176, then 320 patients. CRC screening status was routinely noted in the electronic medical record and CRC screening was implemented in daily routine, increasing eligible patients’ chance to be offered screening. Over a year, screening rates trended upwards, from 37% to 40% (p=0.46) and FIT use increased (2%–7%, p=0.008). Initially, 7/9 PCPs had no patient ever tested with FIT; after the intervention, only 2/8 recorded no FIT tests.

**Conclusions:**

Through data-driven PDSA cycles and significant organisational changes, PCPs of a QC systematically collected data on CRC screening status and implemented SDM tools in their daily routine. This increased patients’ chance to discuss CRC screening. The more balanced use of FIT and colonoscopy suggests that patients’ values and preferences were better respected.

## Problem

Primary care physicians (PCPs) do not usually offer their patients to make an informed choice on colorectal cancer (CRC) screening^1^ and patients might not have the opportunity to decide whether they want to be tested or how they would like to do so. Guidelines emphasise the importance of offering patients a choice between a structural examination like colonoscopy and a high-sensitivity stool-based test like faecal immunochemical test (FIT).^2 3^ When offered both options, patients divide almost evenly in their preferences.^4^ The large preponderance of colonoscopy in Switzerland^5 6^ suggests that PCPs in this country strongly prefer colonoscopy over FIT regardless of patient preferences.^7^ Consistent failure to offer FIT may contribute to low CRC screening rates in Switzerland.^5 8^

We designed this quality improvement (QI) project to encourage practice teams to implement systematic collection of CRC screening status and communication tools promoting shared decision-making (SDM) in CRC screening decisions. We conducted and tested the feasibility of data-driven PDSA cycles in a practice team and aimed to increase the proportion of patients eligible for CRC screening who could express their preference for or against testing and for FIT versus colonoscopy.

## Background

In Switzerland, screening colonoscopies and faecal occult blood tests (FOBTs) such as FIT are nationally reimbursed by the basic health insurance, for people aged 50–69 years. Access to colonoscopy varies by personal insurance plan. If patients are insured under a Health Maintenance Organisation plan, they need to be referred by their PCP. If insured with a flexible plan, patients have direct access to any specialists such as gastroenterologists but are typically referred by their PCP or by other involved specialists. Any specialist can prescribe FOBT, but because PCPs are the coordinating healthcare professionals in Switzerland, they issue almost all the FOBT prescriptions.

CRC kills 1600 people each year and is the third most common cause of death from cancer in Switzerland. If screening with either colonoscopy or FIT begins at age 50, the absolute risk of dying from CRC at age 80 can be halved.^9^ Colonoscopy is accurate but invasive, requires unpleasant preparations that begin the day before, and carries a small risk of serious adverse effects. FIT is convenient, and the patient can take the sample at home. It can detect CRC as well as colonoscopy but may not identify as many polyps.^10^ Since these screening options have various benefits and harms with similar expected efficacy, this makes the choice of CRC screening methods a ‘preference sensitive’ situation in which physicians should accept their patient’s judgement about which test is better for them.^11^

SDM is an approach where clinicians and patients share the best available evidence when faced with the task of making decisions, and where patients are supported to consider options, to achieve informed preferences.^11–13^ If SDM was adopted by most Swiss PCPs to discuss CRC with their patients, it could increase the rate of patients getting the choice to be screened and reduce the discrepancy between patient’s preferences and prescribed methods for CRC screening. SDM is based on the assumption that the PCP is the person most trusted by the patients to help them consider and decide on a treatment path that accords with their values and needs.^12 14 15^

Changing PCPs’ routine so that most will offer patients the opportunity to be tested for CRC and the choice between screening tests poses a serious challenge, and the best place to address it may be in a quality circle (QC). QCs are groups of 6–12 healthcare professionals who meet regularly to reflect on their practice and realise multifaceted, step-based interventions for QI.^16 17^ 80% of PCPs in Switzerland regularly attend QCs^18^ . QCs can use data-driven Plan-Do-Study-Act (PDSA) cycles to implement changes in their practices, especially if PCPs actively collect reliable data that inform their efforts.^19 20^ QC participants create new concepts by combining practitioner-based and evidence-based medical knowledge and test these new concepts to implement them into everyday practice.^21 22^

Practice assistants (PAs) can be deployed to help PCPs overcome organisational barriers to implementing systematic SDM for CRC screening. PAs are health professionals who carry out clinical and administrative tasks in PCP practices; they are common in Germany, the Netherlands, the USA and Switzerland.^23 24^ PAs might be able to take over some work of SDM, lowering the burden that it imposes on PCPs.^25 26^

## Measurement

To assist comprehension, please see online supplementary appendix 1. In collaboration with the PCPs of the QC, we developed and adjusted our data collection form, which allows PCPs to systematically and consecutively collect data on 50–75 y.o. patients seen for more than 5 min in a non-urgent face-to-face consultation.^5 6^ To check consecutive enrolment, PCPs reported the number of weeks it took them to collect all the data. PCPs reported the patient’s age (birth year only), sex and previous CRC testing. We categorised previous CRC testing status as no previous testing, colonoscopy within the last 10 years, colonoscopy more than 10 years ago, FOBT within the past 2 years, FOBT more than 2 years ago, other tests and unknown. We collected data on any prior FOBT because FIT was introduced only 4 years ago in Switzerland; it was likely some patients were screened with other types of FOBT like the guaiac-based test. If patients had been tested within recommended intervals, PCPs did not need to collect more data. If patients had not been tested before or within recommended intervals, PCPs reported if patients had contraindications to CRC screening (life expectancy <5 years, current severe condition or other); if the patient had a contraindication, the PCP could stop collecting data. For eligible patients, PCPs reported whether they had discussed CRC. If they did not discuss it, they reported the reason: it was not appropriate to discuss screening during this visit; they had already discussed screening with the patient; or the patient had been seen during data collection or for another medical reason. If they discussed screening, PCPs reported the presence of potential symptoms of CRC (bloody stools, abdominal pain, weight loss, change in the bowel habits, others) and risk factors for CRC (personal history of CRC or polyps, family history of CRC or polyps, personal history of Crohn disease or ulcerative colitis or other). If the patient refused screening, the PCP noted the reason: no reason for refusal; patient does not feel concerned; patient fears side effects or complications; financial barriers; or other. Then, the PCP recorded the patient’s choice and the test they planned to take (no screening, colonoscopy, FOBT or other). In the second data collection, we added ‘decision postponed’ to the choices to give patients the chance to think about it before making a decision. PCPs took their first measurement in March 2017 and included 20 consecutive patients. They took their second measurement in May 2018 and included 40 consecutive patients.

10.1136/bmjoq-2019-000670.supp1 Supplementary data

## Design

**Figure 1:**
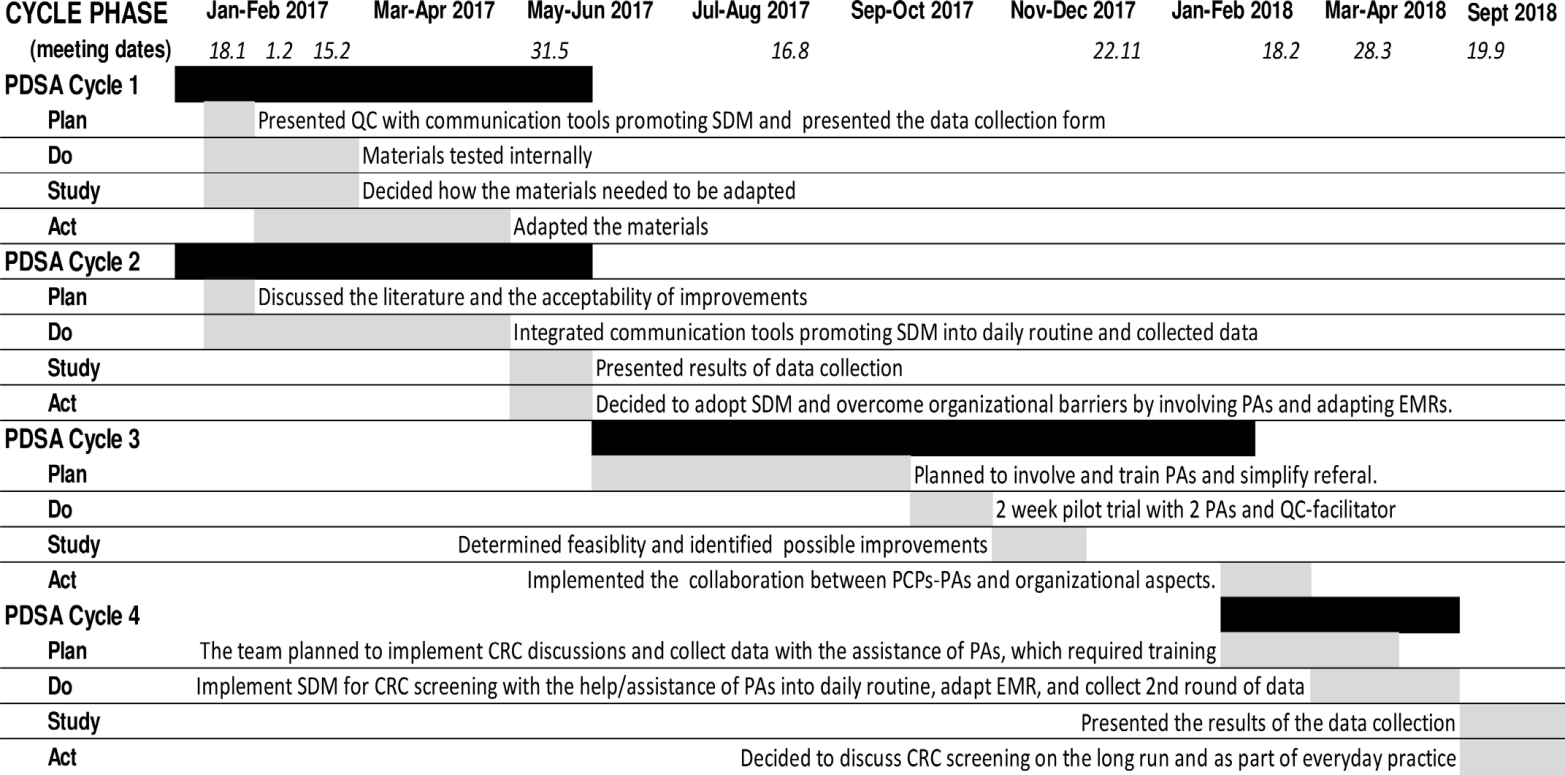
Study timeline.

The long-lasting QC in Wil has been created 21 years ago and currently includes nine PCPs working in the same primary care office. One psychiatrist and one dermatologist working in the same office do not participate to the QC. The group consists of five senior and four younger physicians, five men and four women. They are employed by a large network providing primary healthcare. The patient population is mixed, rural and urban, as the practices serve a small city, surrounding villages and rural areas. Between January 2017 and May 2017, two clinician-researchers from the research team of the Institute of Primary Healthcare of Bern met with the QC’s participants in four QC meetings. PAs from the primary care office participated in the second part of the QI project lasting until September 2018. We describe these meetings in detail in the Strategy section and a timeline (see figure 1) offers an overview of the QI project. One PCP who moved to a different region did not participate to the second data collection.

## Strategy

The research team helped the QC conduct overlapping PDSA cycles.

### PDSA cycle 1 (January 2017–March 2017)

This first PDSA cycle focused on updating existing intervention material consisting in communication tools promoting SDM^15^ to suit QC’s specifications and values. In Switzerland, all citizens have healthcare coverage by law, but flexible deductibles are applied and can be as high as 2500 Swiss franc (≈US$2500). The material presented at the first meeting had been developed for Canton Vaud’s organised CRC screening programme, which waives the deductible of the screening tests and the medical consultation when a test is performed within the programme. Our study was conducted in Canton St Gallen, which has no organised screening programme and where costs of both tests are reimbursed, but deductible is not waived. The QC examined the communication tools which consisted in (1) a two-page structured evidence summary on CRC screening and information about colonoscopy and FIT, (2) a patient decision aid (20-page booklet) on CRC and (3) a four-page abridged version of the booklet to help PCPs discuss CRC screening with patients (‘Decision Board’).^27 28^ The QC also looked at a data collection form developed for this study by the research team.^5 6^ The PCPs studied and discussed the documents and suggested adaptations to better meet their specific needs. The research team updated the materials and data collection process accordingly.

### PDSA cycle 2 (January 2017–June 2017)

This second cycle was the core of the QI project. At the first QC meeting, the research team worked with QC participants to identify and analyse current problems in CRC screening. The group acknowledged current literature on CRC and screening methods. PCPs accepted that screening rates were generally low and that PCPs' preferences for screening method is likely to have a disproportionate influence on the ratio of colonoscopy to FOBT. Participants then set out to change behaviour and use SDM to give their patients an opportunity to discuss CRC screening options. The group used the adapted material in role plays to improve their communication skills. The QC addressed organisational barriers by adjusting their electronic medical record (EMR) and coding patients’ CRC status as well as the performed test. In the next QC meeting, the group agreed that a data collection was necessary to quantitatively assess the current situation using the developed data collection form. The group decided that each PCP should collect data from 20 patients within a 4-week period, and they did this in March 2017. Then, the research group analysed the data and presented them to the PCPs at a fourth QC meeting. The PCPs said they had changed their behaviour and that it took less time to discuss screening with their patient than they had expected. They decided to further implement SDM and suggested they include PAs in the process because these showed interest during the first data collection and their involvement could increase the chance for patients to be screened for CRC.

### PDSA cycle 3 (March 2017–May 2018)

In the third PDSA cycle, PCPs decided to involve PAs and delegate them CRC screening discussions with patients at average risk. PCPs decided to integrate CRC screening into the basic preventive care services the practice regularly provide for patients aged over 50 y.o. In this practice, PAs regularly collect patients’ smoking status and systematically measure their body mass index (BMI), heart rate and blood pressure (BP). The PCPs conducted an internal pilot trial and involved two PAs in SDM about CRC screening options. During a session, the QC facilitator taught the PAs how to use the adapted SDM tools (the booklet intended to patients and the decision board to support discussions on screening). The PAs then practised by role playing. The PCPs implemented a rigorous process that identified high-risk patients; each morning before the consultations, PAs and PCPs briefly discussed the scheduled patients to assess to determine which of the 50–75 y.o. ones were at increased risk for CRC (personal history, familial history or symptoms suggestive for CRC) and if those conditions precluded screening (eg, presence of malignancies or psychiatric diseases). The PCPs discussed screening options with high-risk patients, and the PAs discussed screening with all other eligible patients, if time allowed. If the PA identified CRC risk factors or symptoms during the discussion, the PCPs took over. If a patient opted for FIT, the PA supplied the necessary material and information. If a patient opted for colonoscopy, the PCP performed a few medical tests and organised the referral to the gastroenterologist. After their discussion with eligible patients, PAs coded patient decisions: not willing/contraindication to screening; FIT; colonoscopy; or postponed decision. The new process was tested with two PAs for 2 weeks and then PCPs’ and PAs’ suggestions were integrated; for instance, they simplified the referrals to gastroenterologists. The two PAs found this new process feasible and acceptable. They shared their experiences with the whole PA team during a meeting led by the QC facilitator. Finally, all PCPs met with the two PAs to finalise details before the strategy was routinely implemented.

### PDSA cycle 4 (June 2017–September 2018)

The fourth PDSA cycle evaluated PAs participation in the SDM process. All PCPs and all PAs were invited to a new QC meeting where the majority agreed that the whole team should implement the new process developed in PDSA cycle 3. The PCPs shared evidence and knowledge about CRC and screening with the PAs. They taught them the use of the information booklets and decision boards for SDM through role play. The QC facilitator along with the two PAs involved in the previous cycle presented the morning triage process, where eligible patients were identified. They also introduced electronic reporting of patients’ CRC status as follows: contraindications; chosen screening method; refusal or (in the second data collection round) postponed decision. The QC facilitator and the two PAs established routines to provide patients who chose FIT with information and sampling kits. Patients who chose colonoscopy had blood drawn to exclude a higher bleeding risk and were referred to the closest hospital. The PAs implemented the new process in their routine between January 2018 and April 2018. They discussed CRC screening with patients who were not at high risk whenever possible and used the EMR to report on patients’ CRC status, BMI, heart rate, BP and smoking status. Once this process was embedded in the PAs’ routine (April 2018), PAs and PCPs decided to conduct the second data collection. For this second data collection, the collection form was filled by PAs for non-high-risk patients, and by PCPs for the other ones. The research team met with all PAs at a supplementary meeting and explained how to use the data collection form.

## Results

### PDSA cycle 1

PCPs suggested adapting the intervention documents, notably concerning information on reimbursement of screening tests in their canton, since St Gallen does not have an organised screening programme. They also changed the order of items on the data collection form so it better matched the order in which topics were raised by PCPs when they discussed CRC screening with their patients.

### PDSA cycle 2

**Figure 2:**
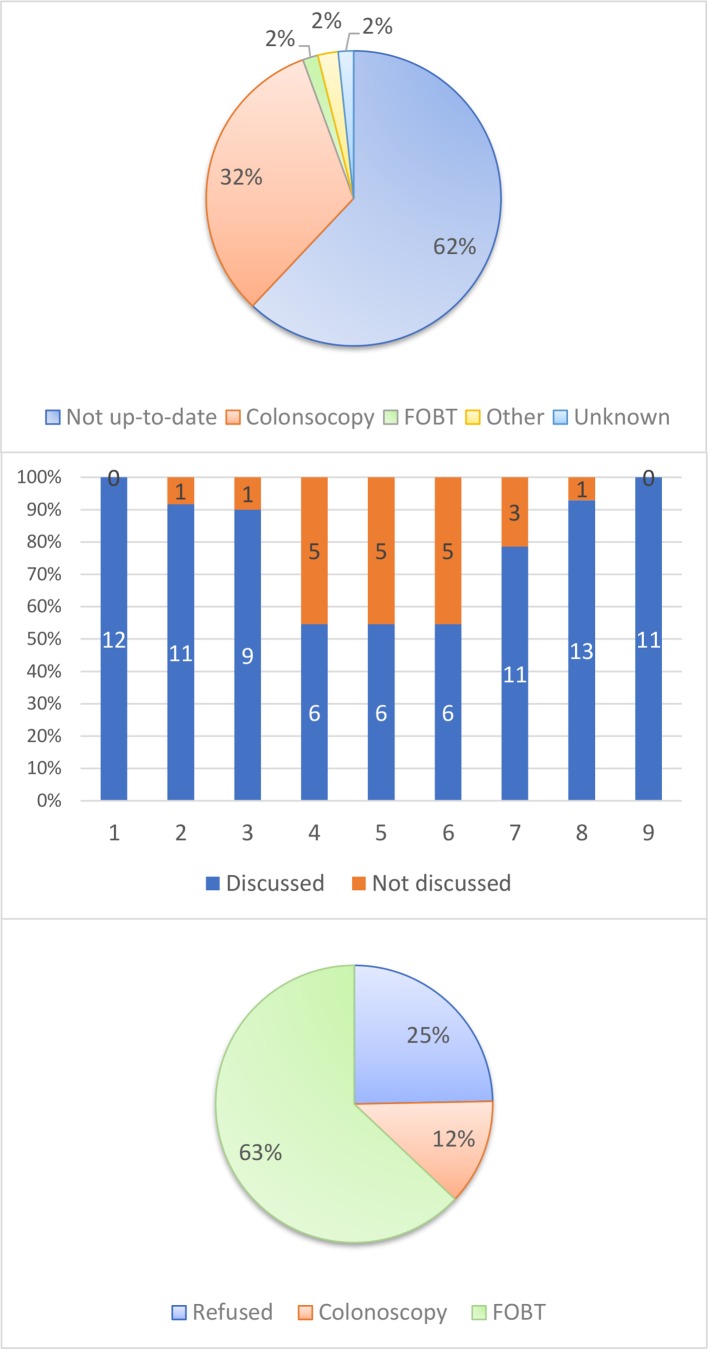
Panel A: CRC status at baseline in 2017 (n=176), Panel B: discussion rates for each PCP in 2017 (n=106), Panel C: decisions after discussion in 2017 (n=85). CRC, colorectal cancer; FOBT, faecal occult blood test; PCP, primary care physician.

PCPs implemented SDM in discussions about CRC screening options and continued to improve their communication techniques. They developed and implemented an electronic coding algorithm ([0;1;2]: refusal/contraindication; colonoscopy <10 years; FIT<2 years) that allowed them to reliably report CRC status. During the first data collection, the nine QC participants reported data on 20 consecutive patients (176 patients; one PCP only collected data on 16 patients). At baseline, screening rate was 37% (65/176) and almost all patients who were already up-to-date (colonoscopy ≤10 years or FOBT ≤2 years) had been screened with colonoscopy (colonoscopy 58/65; FOBT 3/65; other 4/65; see figure 2—Panel A). Of the nine PCPs, seven had no patients ever tested with FOBT (results not shown). PCPs could discuss screening options with most of the previously untested patients (55%–100%) who had not been excluded for contraindications (figure 2—Panel B). After discussion, a quarter of patients refused screening. 63% opted for FIT and 12% opted for colonoscopy (figure 2—Panel C). PCPs found they were able to discuss CRC screening with most eligible patients and it was much less time-consuming to diagnose patients’ preferences than they had expected. But because of some very high discussion rates (100%), the group agreed at a further QC meeting that PCPs could not be expected to discuss CRC screening with all eligible patient they saw.

### PDSA cycle 3

Two PAs learnt to prepare the consultation agenda with PCPs so they could identify patients who were not at high risk, and then used the intervention material to facilitate SDM with these patients. They adapted them to suit their preferences and added the item ‘postponed decision’ to the data collection to capture this common choice. PAs learnt how to use the EMR to report a patient’s CRC status [(0;1;2;3): refusal/contraindication; colonoscopy <10 years; FIT <2 years; decision postponed). Finally, they learnt how to systematically assess CRC screening status and then discuss CRC screening with non-high-risk patients using SDM material.

### PDSA cycle 4

**Figure 3:**
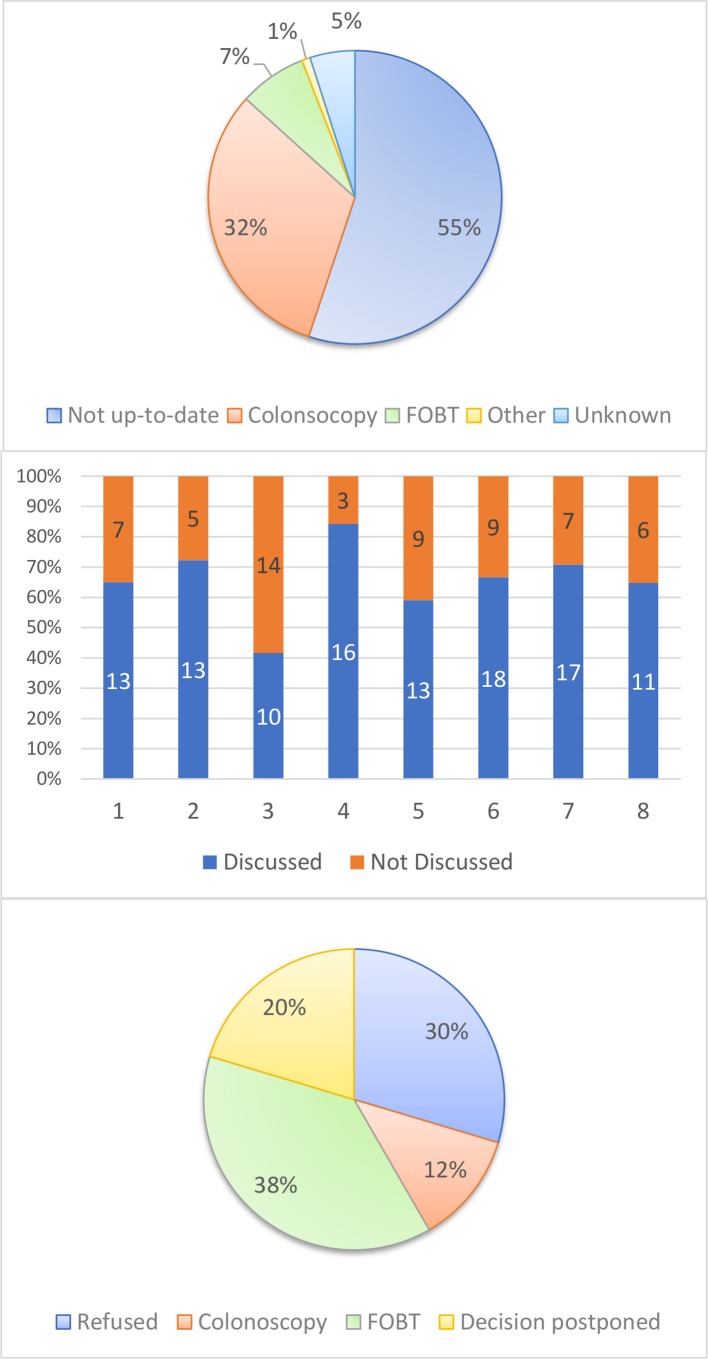
Panel A: CRC status at baseline in 2018 (n=320), Panel B: discussion rates conducted by PAs and PCPs for each PCP in 2018 (n=171), Panel C: decisions after discussion in 2018 (n=111). CRC, colorectal cancer; FOBT, faecal occult blood test; PAs, practice assistants, PCP, primary care physician.

The team (24 PAs and 8 PCPs) participated in data collection and reported data on 40 consecutive patients for each PCP (320 patients). We show our main results in figure 3 (panels A, B and C). Baseline screening rate increased from 37% to 40% but the increase was not statistically significant (p=0.46). The proportion of patients screened with FIT increased more than threefold (from 2% to 7%, p=0.008). The number of PCPs who had no patients tested with FOBT at baseline dropped to 2 of 8 (it was 7/9 in 2017, results not shown). All but one PCP were able to discuss CRC screening with most of their eligible patients (42%–84%). Of patients with whom screening was discussed, 20% decided to postpone their decision and 80% made one (30% refused screening, 38% opted for FIT and 12% for colonoscopy).

### Further results

- PAs added CRC status to routine collection of BMI, BP, and smoking status in EMR.
- PCPs developed routines for colonoscopy referrals that included sampling the patient’s blood, supplying detailed information about the test and giving the patient the necessary instructions for using laxative and dietary instructions.

### Lessons and limitations

When PCPs get the chance to choose what they want to address and can adapt the study material to do so, they actively get involved in the conception of a QI project. They are even able to overcome structural and organisational barriers such as patients’ triage for discussions on CRC screening, time restrains, delivery of quality information and patient referrals.

Baseline screening rates tended to be higher over time (37% vs 40%) and the proportion of patients screened with FOBT increased more than threefold (from 2% to 7%, p=0.008). The number of PCPs who had no patients tested with FOBT dropped from 7/9 in 2017 to 2 of 8 in 2018. These results suggest that PCPs massively changed their prescription patterns for CRC screening while patients could better choose the option which best fitted their preferences.

When PCPs could discuss CRC screening, the vast majority of their patients took an active decision for CRC screening (for or against screening, and with which test). These results suggest the importance and relevance of offering patients a choice. When offered a choice, the proportion of patients who choose either colonoscopy or FOBT does not seem to match the proportion of colonoscopy screenings (89%) found in a recent Swiss study,^5^ which suggests that PCP preferences overdetermine the choice of test.

We found that during first data collection (PDSA cycle 2), some of the PCPs discussed screening with 100% of their eligible patients. They figured out, after discussing this point at a further QC meeting, that it was not practically feasible to do so in a real-life practice. This rate markedly decreased in the second data collection corresponding to what seems to be routinely feasible and reasonable.

Consistent with the literature,^29–31^ participating PCPs found out that they needed to make substantial organisational changes and involve PAs to implement SDM and facilitate discussions on CRC screening. PAs could significantly reduce PCP workloads^25 26^ by identifying and discussing CRC screening with non-high-risk patients, as well as reporting CRC status in the EMR.

The higher screening rates and substantial increase in the number of patients, who had been tested with FOBT after the second data collection, suggest that implementing SDM increased the number of patients who were able to choose which test best suited their preferences and values. We are following up this pilot study with two randomised controlled trials in primary care practices^32 33^ aimed at generating more evidence that training PCPs in SDM can decrease the variation in CRC test choice between PCPs and increase the variation in screening methods,^15^ and that this will ultimately raise screening rates. Our study has some limitations. Since we did not perform a chart-based validation of the data collected, we could not determine the accuracy of PCP self-reports and cannot rule out the possibility of selection bias in choice of participants by PCPs who might not always have followed the rule to include patients consecutively. During the first data collection some PCPs thought the goal was to discuss CRC screening with all eligible patients, which might have influenced and increased prescription rates (after discussion) of the first results. In the second data collection, discussion rates decreased showing how PCPs and PAs integrated the notion that it was not feasible to discuss screening with all patients. Furthermore, our ability to compare the results of the first and second data collection is limited because PCPs alone collected the data in the first round, and PCPs and PAs both collected the data in the second round. Another point needs to be voiced as a limitation; although PCPs changed their practices after the intervention, screening rates at baseline need years to actually represent current prescription practices. The short observation time could explain the only moderate increase in screening rates at baseline between data collections. Finally, although the overall baseline testing rates and test proportions we found seem consistent with the literature,^5 8 34^ generalisation to practices in Switzerland is difficult due to the local context and the limited number of patients assessed in this pilot study.

Our study was strengthened by the use of a data collection form created by PCPs, who made sure it was clear and easy to integrate into their daily routine. Developing the implementation in a QI group allowed us to closely collaborate with PCPs, and allowed the PCPs to work together to make necessary modifications to the intervention materials and the data collection form. This certainly enhanced an important sense of ownership among the participants regarding the study and its material. Furthermore, the study design offered PCPs to set convenient times for meetings, to improve data collection efficiency and PAs involvement as well as to implement changes to the EMR. Piloting the intervention as part of a pragmatic real-life study allowed us to test its suitability for implementation in primary care practices based on collection and analysis of reliable data collected through data-based QC with PDSA cycles. This participatory research study enabled durable changes by increasing acceptance of the intervention by its participants. Furthermore, systematic report of CRC status in the EMR next to BMI, heart rate, BP and smoking status enables simple identification of patients not up-to-date with CRC screening on the long run and helps to ensure sustainability of this project.

PCPs are in a unique position to collect valuable data on discussion and refusal rates, which cannot be gathered by other health care professionals, stakeholders, or insurance companies. Since QC participants could decide which aspects of quality of care are most important and how to improve them, our study presented an exceptional opportunity to measure what they are effectively doing to reach their goal.

## Conclusion

In this pilot QI project, characterised by data-driven PDSA cycles, a QC of PCPs routinely implemented systematic data collection on CRC screening status and communication tools promoting SDM in CRC screening decisions. Combining a multilevel training intervention with significant organisational changes such as involving PAs, the QC of PCPs successfully increased the chance for eligible patients to be offered to be screened for CRC and to choose between CRC screening options. The use of FIT increased more than threefold, which indicates that a higher proportion of patients might have been able to choose the test that best matched their preferences and values.
