# Supplementary figures and images for: Offering patients a choice for colorectal cancer screening: a quality improvement pilot study in a quality circle of primary care physicians
Source: BMJ Open Qual. 2019 Oct 3;8(4):e000670. doi: 10.1136/bmjoq-2019-000670 (PMC6797289; doi:10.1136/bmjoq-2019-000670)

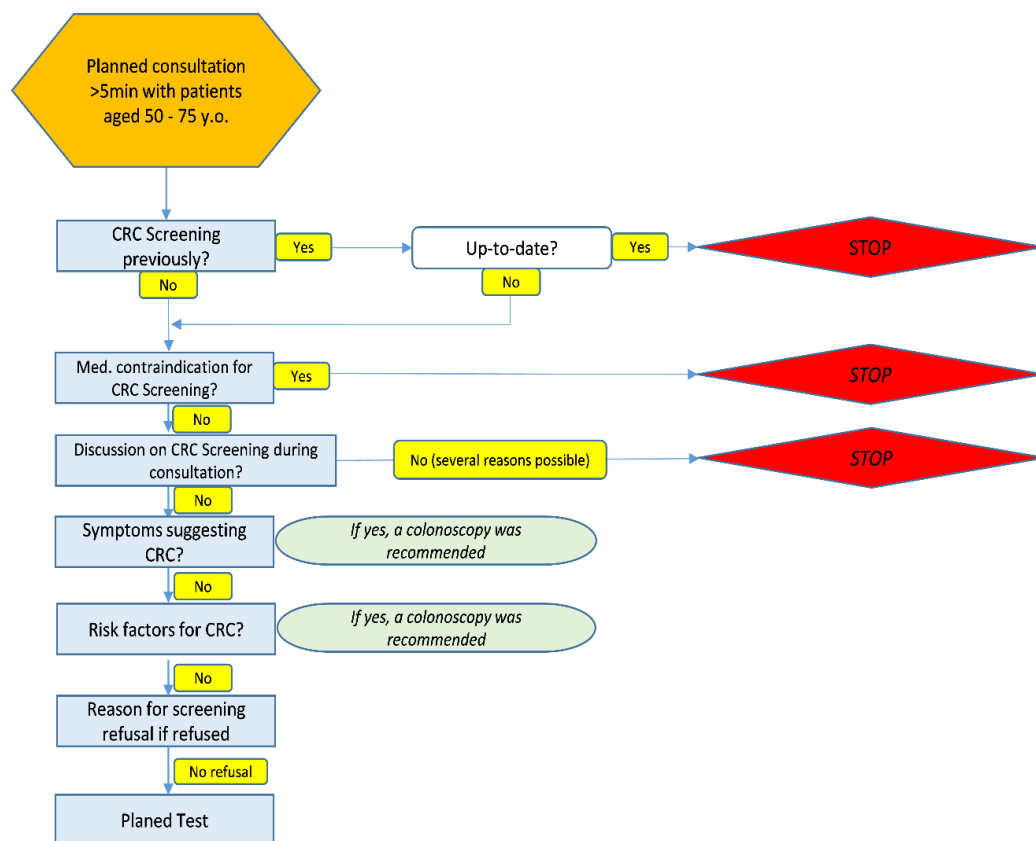

Supplement: Supplementary data [file bmjoq-2019-000670supp001.pdf]
